# Fall‐armyworm invasion, control practices and resistance breeding in Sub‐Saharan Africa

**DOI:** 10.1002/csc2.20317

**Published:** 2020-11-11

**Authors:** Prince M. Matova, Casper N. Kamutando, Cosmos Magorokosho, Dumisani Kutywayo, Freeman Gutsa, Maryke Labuschagne

**Affiliations:** ^1^ Department of Research and Specialist Services Crop Breeding Institute 5th Street Extension Harare Zimbabwe; ^2^ Department of Crop Science University of Zimbabwe Harare Zimbabwe; ^3^ International Maize and Wheat Improvement Centre Harare Zimbabwe; ^4^ Department of Plant Sciences University of the Free State Bloemfontein South Africa

## Abstract

Fall armyworm [*Spodoptera frugiperda* (J.E. Smith); FAW] invasion has exacerbated maize (*Zea mays* L.) crop yield losses in sub‐Saharan Africa (SSA), already threatened by other stresses, especially those that are climate‐change induced. The FAW is difficult to control, manage, or eradicate, because it is polyphagous and trans‐boundary, multiplies fast, has a short life cycle and migrates easily, and lacks the diapause growth phase. In this study, FAW and its impact in Africa was reviewed, as well as past and present control strategies for this pest. Pesticides, cultural practices, natural enemies, host‐plant resistance, integrated pest management (IPM), and plant breeding approaches were examined as possible control strategies. It was concluded that an IPM control strategy, guided by cultural approaches already being used by farmers, and what can be adopted from the Americas, coupled with an insect‐resistance management strategy, is the best option to manage this pest in Africa. These strategies will be strengthened by breeding for multi‐trait host‐plant resistance through stacking of genes for different modes of control of the pest.

**Abbreviations**
*Bt*: *Bacillus thuringiensis*
CIMMYT: Centro Internacional de Mejoramiento de Maíz y Trigo (International Maize and Wheat Improvement Center)
CML: CIMMYT maize line
EIL: economic injury level
ETL: economic threshold level
FAO: Food and Agriculture Organization
FAW: fall armyworm
GM: genetically modified
ICIPE: International Centre of Insect Physiology and Ecology
IITA: International Institute of Tropical Agriculture
IPM: integrated pest management
OPV: open‐pollinated variety
PPT: push–pull technology
SSA: sub‐Saharan Africa
USDA‐ARS: U.S. Department of Agriculture ‐ Agricultural Research Service
WEMA: water efficient maize for Africa.

## 1 INTRODUCTION

The sub‐Saharan Africa (SSA) region is dominated by smallholder farming systems characterized by intense cereal crop production, maize [*Zea mays* L.] being the most important staple crop for humans and feed crop for livestock (ACAPS, 2017; Smale et al., 2011; Tambo et al., 2019). More than 300 million farming families depend on maize for food security and livelihoods in Africa (Cock et al., 2017; Kumela et al., 2019; VIB, 2017). Maize production in SSA is dominated by smallholder farmers (CIMMYT, 2019; Edmeades et al., 2017; Sisay et al., 2018), growing the crop under diverse climatic and socio‐economic conditions (Cairns et al., 2013). Climate‐induced maize production constraints include heat and drought stress (Cairns et al., 2013), and emerging invasive insect pests and diseases (CIMMYT, 2019; IITA, 2019). The socio‐economic factors affecting productivity of maize include small pieces of land, unaffordable input costs, limited labor, and lack of machinery to ease farming operations (VIB, 2017). As a result of the above constraints, maize yields in Africa are generally low, averaging around 2.0 Mg ha^−1^ or below (Cairns et al., 2013; Edmeades et al., 2017; VIB,  2017).

Fall armyworm [*Spodoptera frugiperda* (J.E. Smith); FAW] is currently the most damaging crop pest affecting maize in SSA, where it has spread very widely (ACAPS, 2017; Day et al., 2017; Kumela et al., 2019). It is a polyphagous (feeds on several hosts) and migratory (can spread to other countries) pest that survives on at least 80 plant species, including maize, wheat (*Triticum aestivum* L.), sorghum [*Sorghum bicolor* (L.) Moench], and rice (*Oryza sativa* L.) (Harrison et al., 2019; Prasanna et al., 2018; Sibanda, 2017). In the past, the pest was mainly found in North and South America where it is the most important pest of maize (Hruska, 2019; Pannuti et al., 2016), but recently, its two strains, the rice strain (R‐strain) and the corn strain (C‐strain), invaded new territories in the tropical and subtropical African regions (Cock et al., 2017; FAO, 2019a; Fatoretto et al., 2017). The two strains are morphologically identical but different in physiological features and host‐plant preferences (Nagoshi et al., 2018; Nagoshi & Meagher, 2018). The R‐strain prefers smaller grass species (especially millet) and pasture habitats, whereas the C‐strain prefers larger grasses, such as maize and sorghum (Nagoshi & Meagher, 2018). Fall armyworm was first reported in Africa in 2016 (Goergen et al., 2016; Cock et al., 2017); and since then, it has spread throughout SSA and Asia, causing significant crop damage resulting in economic losses (Abrahams et al., 2017; Rwomushana et al., 2018; Tambo et al., 2019). Evidence suggests that the races introduced in Africa originated from southern Florida and the Caribbean (Nagoshi et al., 2018). The consequences of FAW invasions on food and nutrition security in SSA have been made worse by lack of resistant/tolerant cultivars, poor capacity to control and manage the pest (Day et al., 2017; Harrison et al., 2019), and the suitability of the climatic conditions for the rapid multiplication and perpetuation of this pest (Prasanna et al., 2018). Fall armyworm, riding on migratory winds, has the potential to travel for long distances, and can prolifically breed in suitable environmental conditions typical of SSA (Abrahams et al., 2017; Baudron et al., 2019; Kumela et al.,  2019).

**Core Ideas**

- Fall armyworm is a devastating pest on maize in Africa.
- Control strategies include pesticides, cultural practices, natural enemies, IPM, and resistance.
- The IPM and host plant resistance are the most appropriate control strategy.
- Breeding for multitrait host plant resistance is the best long‐term control strategy.

Currently, researchers are working on immediate and long‐term solutions to the problem. Breeders are developing cultivars that can offer native resistance to the pest, while chemical companies, entomologists, and other researchers are developing insecticides, bio‐controls, and cultural‐methods, respectively, to minimize crop damage that can result after infestation (Wightman, 2018). Most farmers are relying on mechanical control methods, indigenous and farmer‐to‐farmer advice, and recommendations from extension services (Baudron et al., 2019; Kumela et al., 2019). Mechanical control methods are those cumbersome approaches that include hand crushing of the larvae, moths, and eggs; placing sand and ashes in leaf whorls; and other cultural practices. Baudron et al. (2019) viewed such practices as labor intensive, exhaustive, time‐consuming, and not practical, especially for women, who are usually the dominant labor force in smallholder agricultural systems. Research and extension are advising farmers to use IPM to manage the pest. This is targeted at minimizing chemical damage to people and the environment, while targeting effective pest control (Day et al., 2017). In this review, the aim is to provide: (a) a summary of the current and the potential negative effects of FAW invasion on the livelihoods of smallholder communal farmers in SSA, (b) a synthesis of past and present approaches targeted at managing this pest, and (c) the prospects for developing FAW‐resistant maize genotypes for SSA. The research questions guiding the review included: (a) the current and anticipated FAW impact in SSA, (b) the management of the pest by smallholder farmers, and (c) possible best management practices and strategies suited for SSA.

## 2 THE PEST, ITS MIGRATION INTO AFRICA, AND ITS IMPACT

### 2.1 Morphology and biology of fall armyworm

**FIGURE 1:**
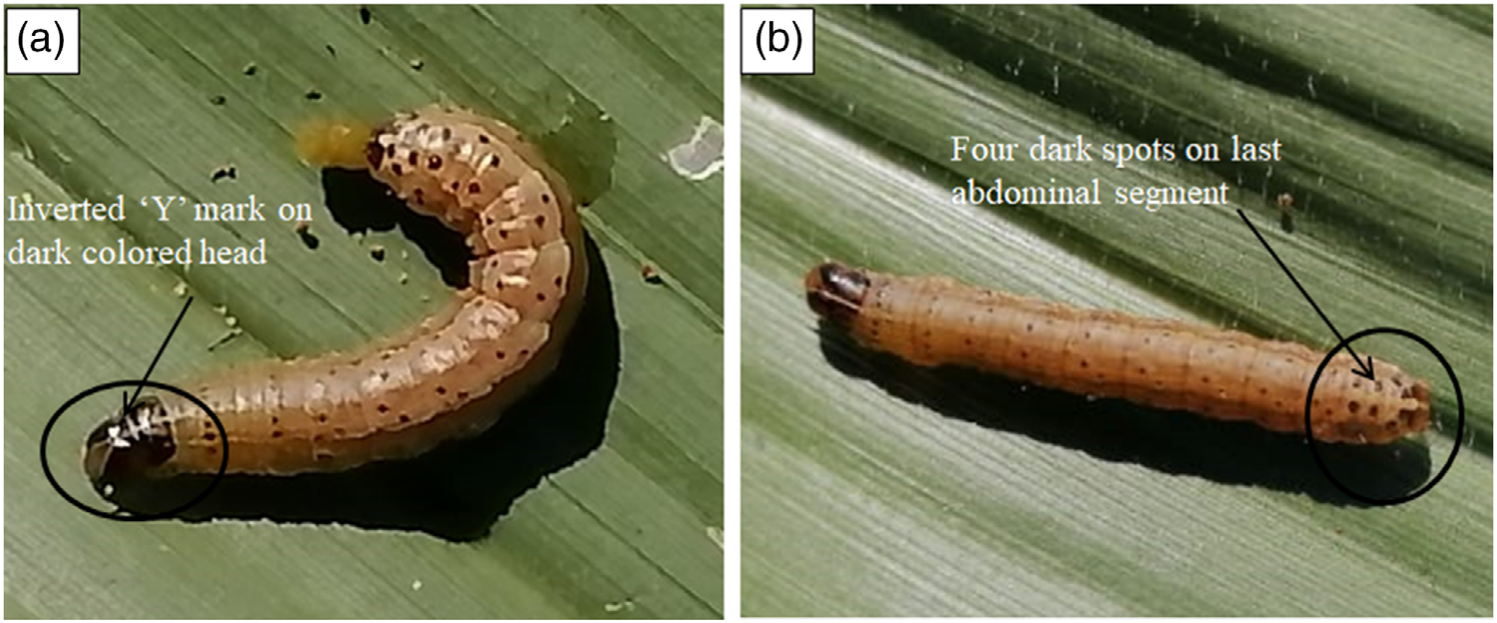
Physical appearance of the fall armyworm (FAW) larvae highlighting the most distinguishing features of the worm (photograph produced by Stanley Gokoma, edited by Prince M. Matova)

Fall armyworm resembles both the African army worm [*Mythimna unipuncta* (Haworth)] and corn earworm [*Helicoverpa zea* (Boddie)]. However, FAW has some distinct features that can help separate it from its close relatives (Prasanna et al., 2018). These include: (a) a white‐colored inverted “Y” mark on the front of the dark head and (b) a brown head with dark honey‐combed markings (Figure 1a) and, (c) four dark spots displayed in a square on top of the eighth abdominal segment (Rwomushana et al., 2018; Prasanna et al., 2018), as shown in Figure 1b.

**FIGURE 2:**
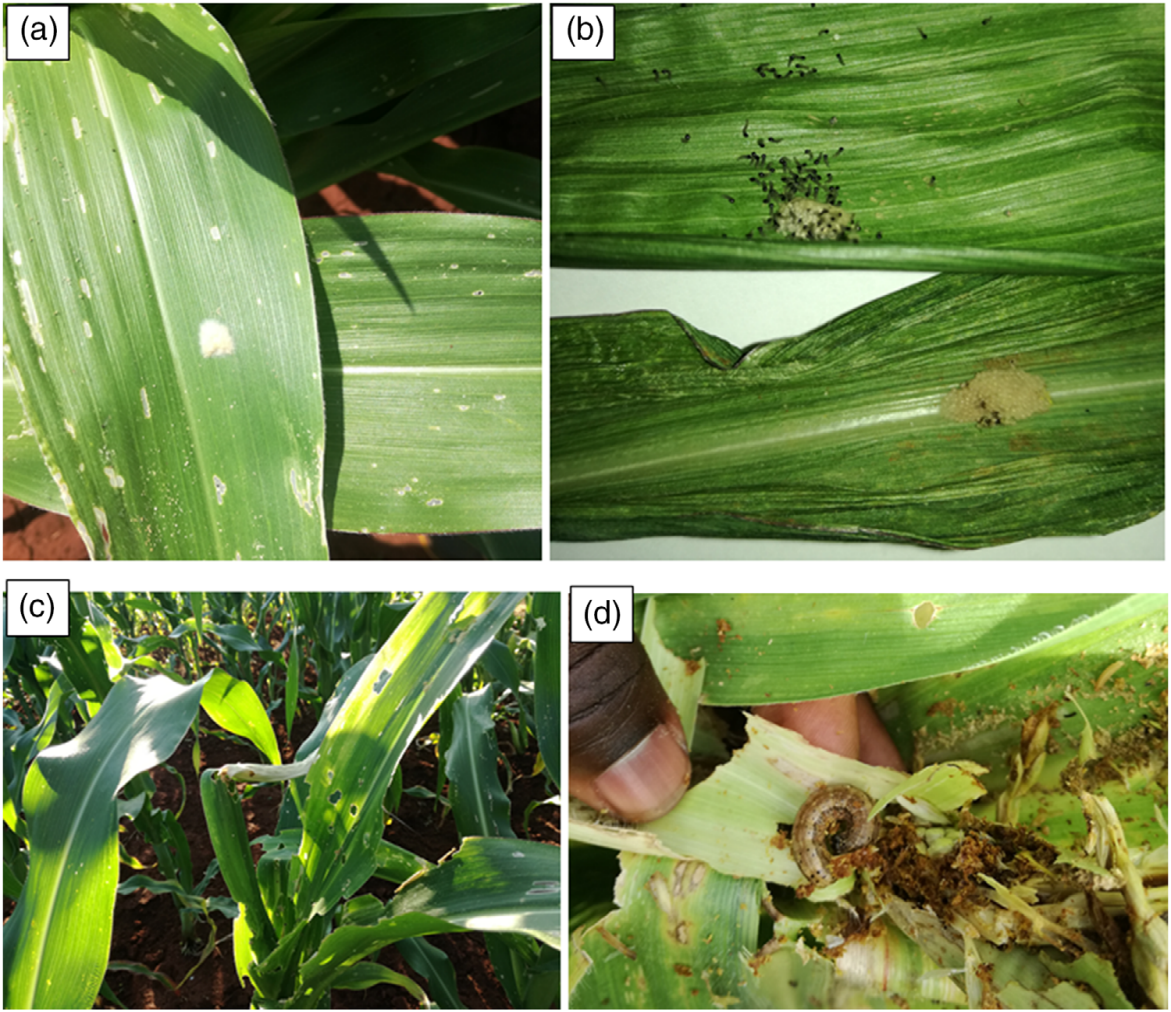
(a) Fall armyworm (FAW) egg masses and first signs of FAW infestation on leaves, (b) Young FAW larvae (black heads) emerging from egg masses on window pane damaged leaves, (c) Advanced FAW damage, showing dead heart on the growing point, (d) Large FAW larvae protected by ‘frass plug’ while feeding in the whorl during tasseling stage (photographs produced by Prince M. Matova)

Typical early FAW infestation signs and symptoms include small “pin holes” and “window panes” (Figure 2a), resulting from feeding of the small worms on leaves (Figure 2b). Damage of maize plants caused by FAW attack is severe during the late pre‐tassel stage (Figure 2c). Bigger larvae consume large amounts of tissue and do much more damage compared to small larvae, resulting in a ragged appearance of the leaves (Figure 2d; Prasanna et al., 2018). It is also important to appreciate that foliar damage on maize may look serious but may not necessarily translate into high grain yield losses (Hruska, 2019; Wightman, 2018). Hruska (2019) reported a study carried out by the U.S. Department of Agriculture ‐ Agricultural Research Service (USDA‐ARS), in which they noted that FAW defoliation as high as 70% at 12‐leaf stage could cause just about 15% grain yield loss. Fall armyworm defoliation on maize rarely goes above 50% (Hruska,  2019).

The FAW's rate of reproduction and multiplication is rapid. For instance, one adult female moth is capable of laying between 1,000−2,000 eggs during its lifetime (Hruska, 2019). Eggs are laid in egg masses of between 100−200 eggs (Figure 2a and 2b). In warmer climates, the duration of the egg stage is only 2−3 d (Hruska, 2019). The larval stage lasts between 14 and 30 d in warmer summer and cooler winter months, respectively (Kumela et al., 2019; Pitre et al., 1983), whereas the lifespan of an adult moth is approximately 10 d. The pest is able to complete its life cycle in 30 d at an average daily temperature of 28 °C (ACAPS, 2017; Prasanna et al., 2018). This implies that in warm climates, such as those experienced in SSA, FAW can have multiple generations in one season (Nboyine et al., 2020). Coupled with the fact that FAW does not have a diapause (the biological resting period), it can establish as an endemic pest (Harrison et al., 2019; Hruska,  2019).

### 2.2 Introduction and spread of fall armyworm in sub‐Saharan Africa and beyond

The FAW is native to the western hemisphere (particularly North and South America) (Pannuti et al., 2016; Sisay et al., 2018), where the insect has been a problem pest in crops for several decades (FAO, 2017; Fatoretto et al., 2017; Hruska, 2019). Several reports confirmed that FAW was initially established in São Tomé and Príncipe, Benin, Nigeria, and Togo in 2016 (Nagoshi et al., 2018; Nagoshi & Meagher, 2018; Prasanna et al., 2018). Fall armyworm is thought to have come into Africa through stowaways on commercial aircrafts, in cargo, or airplanes (Day et al., 2017). As of December 2018, the trans‐boundary pest was reported to be present in almost all SSA countries (FAO, 2019a). The pest was detected in Yemen and India in August 2018 (FAO, 2019a; Tambo et al., 2019). As was predicted by modeling (Day et al., 2017), FAW has spread to all of the SSA countries, parts of the Middle East and Asia (Hruska, 2019; Tambo et al., 2019; Wightman, 2018), and there are chances that the pest will spread to Europe. The spread is suspected to be attributable to natural migration and through trade (Baudron et al.,  2019; Day et al., 2017).

### 2.3 Impact of fall armyworm in Africa

Fall armyworm is likely to fully establish and continue to cause maize crop losses in the region because of the suitability of the region's climate for the pest's growth and development (Baudron et al., 2019). Additionally, its short life cycle, ability to travel across large geographical areas, and its wide host range promote its rapid multiplication and spread, making it difficult to control (Day et al., 2017; Prasanna et al., 2018). The African Union's sustainable development goal number 7 (G7), targeted at reducing poverty and hunger, may be difficult to achieve in the absence of effective FAW‐management strategies (Harrison et al., 2019). Fall armyworm is envisaged to be impacting negatively on maize crop production, maize seed production, human health, environmental integrity, and maize trade with regions where the pest has not been reported.

#### 2.3.1 Impact on crop production

Available evidence suggests that FAW is difficult to control or manage (Day et al., 2017; Prasanna et al., 2018; Santos‐Amaya et al., 2016). This is primarily because of its feeding and sheltering behavior on the host plant. When FAW invaded Africa in 2016, the first affected farmers suffered huge losses because of unpreparedness. This was confirmed by farmers in Zambia, who reported that FAW intensity was less severe in 2017 compared to 2016, and they attributed this to awareness and early response (Kansiime et al., 2019). However, responding with pesticide control reduces profit thresholds for those farmers who grow maize for commercial purposes.

The SSA maize production environment is traditionally burdened by multiple abiotic and biotic stresses (Cairns et al., 2013; CIMMYT, 2019; IITA, 2019). Fall armyworm invasion further worsens the burden on the resource‐poor SSA smallholder farmers (ACAPS, 2017; Wightman, 2018). Kumela et al. (2019) reported that farmers in Kenya and Ethiopia reported yield losses of 0.77−1.0 Mg ha^−1^, attributable to FAW infestation. These farmers reported infestation levels that averaged 32% (Ethiopia) and 47.3% (Kenya) during that season, and anticipated a general trend of increasing infestation in future seasons (Kumela et al., 2019). In maize, FAW feeds on foliage, reproductive parts, and grain. Feeding on foliage reduces economic yields by causing injury to the plant, including dead heart and reducing the photosynthetic area, whereas feeding on reproductive parts inhibits or reduces fertilization and grain formation, hence reduced final grain yield (Tambo et al., 2019). Fall armyworm is also capable of partially or totally devouring maize grain, thereby reducing grain quality and ultimately grain yield (Harrison et al., 2019). In the Americas, FAW infestations have been reported to cause yield losses ranging from 25 to 100%, depending on the severity of infestations (Arias et al., 2011; De Oliveira et al., 2018; Womack et al.,  2018).

Earlier reports on impacts of FAW indicate that maize losses could be higher than previously estimated (Day et al., 2017; Sisay et al., 2018). Yield losses were also anticipated in other major African crops, mainly sorghum, millet, and legume (Harrison et al., 2019; Sibanda, 2017). A survey by the FAO (2018a) in Namibia reported yield losses, attributable to FAW in maize, sorghum, and millet. The survey showed that in 2017, only a small proportion of farmers (17%) engaged in coping strategies following the negative effects of FAW invasion. Another study by Abrahams et al. (2017) estimated potential annual maize losses in 12 African countries at 21−53%, with an economic damage projected at US$2.48−6.19 million. Similar results were obtained by Baudron et al. (2019), from their studies in Chipinge and Makoni districts in Zimbabwe, in which they reported FAW‐caused losses of 11.57 and 16.39%, respectively, for the two districts. The recorded infestation levels ranged between 32 and 48%. A 2018 study by Rwomushana et al. (2018) reported yield losses of 26−40% and 35−50% for Ghana and Zambia, respectively, and recently, De Groote et al. (2020) reported similar yield‐loss levels, averaging 32−34% for Kenya. As Hruska and Gladstone (2014) and De Oliveira et al. (2018) reported, the extent of damage varied with the severity of infestation.

#### 2.3.2 Impact on livelihoods

Yield losses reported by Rwomushana et al. (2018), Baudron et al. (2019), Kumela et al. (2019), and others have significant negative impacts on livelihoods of smallholder farmers, given that maize is a subsistence crop in SSA. Most households, including female‐headed families in SSA, rely on income from subsistence farming to support payment of key livelihood activities, including school fees for children. Using various socio‐economic and agro‐ecological data, the FAO estimated food insecurity levels across households in SSA as a function of exposure to FAW invasion risk, and vulnerability and lack of coping strategies among the exposed populations. The study produced a map that is intended to help identify areas where household food insecurity due to FAW is highest, to assist decision makers in deployment of the needed assistance in the region (http://www.fao.org/emergencies/resources/maps/detail/en/c/1110178/). However, there is a need to carry out additional impact assessment studies on livelihoods of smallholder farmers in SSA to assess the magnitude of the negative impact of FAW on their livelihoods. Even in the absence of such evidence, it is imperative that effective FAW‐management strategies be put in place to guard against the pest's potential negative effects on food security and livelihoods of resource‐poor farmers in SSA (Goergen et al., 2016; Harrison et al., 2019; Tambo et al.,  2019; Wightman, 2018).

#### 2.3.3 Impact on human health, non‐target organisms, and the environment

Use of pesticides to control FAW predisposes farmers to harmful insecticide contamination and also destabilizes the ecosystem by killing non‐target organisms (Kumela et al., 2019; Mihm, 1997). Hence, development of sustainable and environment‐friendly control strategies for FAW is paramount. Initial reports from interactions with farmers and search of the internet have shown that most farmers identify the pest late in their fields and they usually do panic spraying (Day et al., 2017). As a result, they use different control strategies, including indiscriminate pesticide use, which pre‐disposes the farmers, maize grain, livestock, and the environment to risk of contamination (Carvalho et al., 2013; Harrison et al., 2019). Indiscriminate use of pesticides has the disadvantage of killing non‐target insects, including natural enemies of the intended pest and other agricultural pests (Harrison et al., 2019). This disrupts normal ecosystem functioning and may actually promote the perpetuation of the targeted pest and create outbreaks of other pests that were being naturally suppressed by their natural predators and parasitoids.

Recent studies have shown that most farmers are not aware of, or simply ignore, the dangers of insecticides on human health and non‐target insects (Kansiime et al., 2019; Kumela et al., 2019). Day et al. (2017) and Kansiime et al. (2019) reported the use of cheaper, less effective, and moderately hazardous insecticides, which might have negative effects on human health, the environment, and natural enemies of the target and other pests (Sisay et al., 2018). Famers in Zambia were reported to have used Monocrotophos [dimethyl (*E*)‐1‐methyl‐2‐(methylcarbamoyl)vinyl phosphate], a Class 1b (highly hazardous) insecticide according to the World Health Organization classification (Kansiime et al., 2019). More than 50% of farmers in a study by Kansiime et al. (2019) were reported not to be using personal protective equipment during insecticide spraying. Some farmers spray maize during the grain‐filling to maturity stages, scenarios which create the risk of pesticide contamination and residual effect on grain intended for consumption.

Many smallholder farmers in Africa are ignorant of human and environmental safety when using synthetic insecticides (Harrison et al., 2019; Kansiime et al., 2019) and there is conclusive evidence of misuse, resulting in toxicity to humans, reduced efficacy, and development of insect resistance (Sola et al., 2014; Stevenson et al., 2017). This is supported by a report by the United Nations that projected the cost of injury and illness linked to pesticides at $90 billion between 2005 and 2020 (Lewis et al., 2016). The practice of using incorrect application rates also promotes the rapid development of insecticide resistance in the pest (Sisay et al.,  2018).

#### 2.3.4 Impact on seed companies

Farmers in SSA rely mainly on certified maize seed, yet the majority of seed companies supplying most of the seed in a number of countries are still developing (African Centre for Biodiversity, 2015). The sudden emergence of FAW in 2016 and its resurgence and spread in the following seasons may compromise the economic viability of most small and emerging seed companies. This is worsened by the fact that most of these companies produce their seed with small‐scale farmers. Such seed companies may need to invest in extensive grower training on identification and management of FAW.

Studies on smallholder farmers in Kenya and Ethiopia by Kumela et al. (2019), and in Zambia by Kansiime et al. (2019) showed that most farmers were able to observe FAW larvae on infected plants, while a few could identify eggs and adult stages of the pest. This has serious implications for FAW management by farmers, as early scouting and identification of eggs and early larval stages are key to ensuring effective control (Nboyine et al., 2020; Prasanna et al., 2018).

#### 2.3.5 Impact on trade

The pest is likely to affect maize trade, including seed, grain, and green maize between Africa and countries without FAW, because of strict and thorough checking at entry points (ACAPS, 2017). Reports suggest that grain consignments contaminated with FAW, coming from Africa, were prevented from entering Europe (Goergen et al., 2016). Lately, South Africa had started shipping maize grain for stock feed to the European Union (https://www.biznews.com/global-investing/2018/05/23/record-corn-exports-land-debate-key). There are chances that this may not continue. Seed maize export from Africa to areas without FAW is likely going to be difficult or even impossible because of biosecurity measures across regions.

## 3 FALL ARMYWORM CONTROL STRATEGIES: PAST AND PRESENT

Maize and FAW are both native to the Americas. The two have co‐existed in their natural habitat for many years (FAO, 2017). Farmer experiences and research on FAW in maize during the past 100 yr in Mesoamerica have drawn some key lessons that can be used to effectively manage and control FAW in areas where it has recently invaded (Assefa & Ayalew, 2019; Tambo et al., 2019). Traditionally, the Americans have been controlling FAW using host‐plant resistance, insecticides (synthetics and botanicals), cultural practices (including early planting, crop rotations, and intercropping), and IPM approaches (Abrahams et al., 2017; Tambo et al., 2019; Womack et al., 2018). Farmers and researchers in SSA need to adopt and customize some of these strategies to suit the African farming practices according to farm size and various socio‐economic factors (Tambo et al.,  2019; Wightman, 2018).

### 3.1 Factors influencing smallholder farmers’ choice of fall armyworm control strategies

Farmers’ choice of FAW control strategy is affected by various factors, which include availability of a control strategy (including its effectiveness and ease of use) and resources, gender, and age among other issues. Kansiime et al. (2019) implicated farmers’ gender and age as factors affecting control practices; insecticide application was popular among men, and cultural/mechanical methods were mostly practiced by women.

Sola et al. (2014) reported that farmers in Africa found insecticides unaffordable, as a result many farmers avoided using them. Social settings in most of SSA are such that men have better access to, and control of, finances; hence they tend to use that advantage to make work easier for them. This could explain why synthetic insecticide use was dominated by men. Women, because they usually lack financial resources and are the main labor force in the fields, tend to prefer mechanical control methods. Men used combined control practices more than women across ages, while elderly farmers preferred cultural methods, sometimes combined with synthetic and botanical pesticides, compared to younger farmers, who preferred synthetic pesticides (Kansiime et al., 2019). This is probably because young farmers and men in general prefer quick results, which normally come with synthetic insecticides, and they normally have the funds to purchase insecticides. Use of insecticides by elderly farmers may be motivated by age, as they no longer have the strength to use mechanical methods.

Given that FAW is migratory in nature and can quickly build up a population in a short time, pest containment at the community level under the smallholder farmer setup was seen to be a challenge, since control strategies are based on individual farmer preferences (Kansiime et al., 2019). Poorly controlled fields harbor the pest, and this causes new infestations, even in fields where farmers effectively control the pest. Kansiime et al. (2019) regressed grain yield against various socio‐economic and management practices on FAW infestation levels and the results showed a strong positive correlation between grain yield and use of cultural practices and pesticides in managing this problematic pest. This implied that farmers, who tried to control FAW using one or a combination of methods, would be able to secure grain yield from FAW damage.

### 3.2 Fall armyworm control practices

#### 3.2.1 Synthetic and botanical pesticide control practices

Smallholder African farmers rarely use pesticides in maize production. Using data from a survey carried out between 2010 and 2012, Hruska (2019) reported that pesticide use (across all crops) could be as low as 3% in countries without input subsidies, such as Malawi. A 2017 study by the FAO reported that about 1.7% of smallholder farmers in Namibia used pesticides in crop production (FAO, 2018b; Hruska, 2019). In Kenya and Ethiopia, some farmers confused the FAW with some familiar pests such as common African stalk borer (*Busseola fusca*), spotted stalk borer (*Chilo partellus*), or African armyworm (*Spodoptera exempta*), hence they made wrong choices in pesticide application, which did not help control the pest (Kumela et al., 2019). Appropriate insecticides are usually costly, which tends to push farmers to use cheaper alternatives they come across in trying to eliminate this invasive insect pest. Coupled with incorrect application rates and procedures, this could explain why some farmers in Kenya and Zimbabwe felt insecticides were not being effective in controlling FAW (Baudron et al., 2019; Kumela et al., 2019). In contrast, 97% of farmers in Zambia confirmed that pesticide application helped in controlling FAW, especially when the pesticides were alternated (Kansiime et al.,  2019).

As an emergency response strategy to FAW invasion in 2016, most governments in Africa distributed chemical insecticides to farmers through extension. However, most of these were ineffective, since efficacy trials had not been done at that time and the insecticides were used indiscriminately (Day et al., 2017; Sisay et al., 2018). Some of the broad‐spectrum pesticides that were being used included Thionex [Endosulfan 50% (Hexachlorohexahydromethano‐2,4,3‐benzodioxathiepin‐3‐oxide)], Carbaryl [Carbaryl 85WP (1‐naphthyl methylcarbamate)], Dimethoate [Dimethoate 40EC (C_5_H_12_NO_3_PS_2_)], and Karate [Lambda cyhalothrin 5EC (C_23_H_19_ClF_3_NO_3_). These were later replaced by more efficient and eco‐friendly pesticides, which included Ecoterex [Deltamethrin (C_22_H_19_Br_2_NO_3_) and Pirimiphos methyl (C_11_H_20_N_3_O_3_PS)], Emamectin benzoate (4′′‐Deoxy‐4′′‐epi‐methylamino‐avermectin B1)/Macten (Emamectin benzoate 5%), Super dash [Emamectin benzoate and Acetamiprid [N‐(6‐Chloro‐3‐pyridylmethyl)‐N′‐cyano‐acetamidine)], Ampligo [Chlorantraniliprole (3‐Bromo‐4′‐chloro‐1‐(3‐chloro‐2‐pyridyl)‐2′‐methyl‐6′‐(methylcarbamoyl)pyrazole‐5‐carboxanilide, 3‐Bromo‐N‐[4‐chloro‐2‐methyl‐6‐[(methylamino)carbonyl]phenyl]‐1‐(3‐chloro‐2‐pyridinyl)‐1H‐pyrazole‐5‐carboxamide, DPX E2Y45) and Lambda‐cyhalothrin (C_23_H_19_ClF_3_NO_3_)], and Belt [Flubendiamide (N2‐[1,1‐Dimethyl‐2‐(methylsulfonyl)ethyl]‐3‐iodo‐N1‐{2‐methyl‐4‐[1,2,2,2‐tetrafluoro‐1‐(trifluoromethyl)ethyl]phenyl}‐1,2‐benzenedicarboxamide)].

Governments’ intervention promoted the use of insecticides among smallholder farmers in most countries, for instance, it was reported that 72 and 60−62% farmers in Ghana and Zambia, respectively, used inorganic insecticides to control FAW in 2017 (Hruska, 2019; Kansiime et al., 2019). The reported increase in pesticide use among smallholder farmers was motivated by the availability of free insecticides distributed by national governments (Day et al., 2017; Kansiime et al., 2019). In those areas where insecticides were not distributed, most farmers simply did not know what to use to control the pest, and some farmers did not use chemical pesticides because of their high cost; as a result farmers suffered yield losses without any control intervention (FAO, 2018b; Rwomushana et al., 2018).

Chemical insecticide control for FAW is a common practice in North and South America (Fatoretto et al., 2017). In contrast, most smallholder farmers in Africa, where there is no government support, cannot afford the repeated spraying required to achieve effective control (Sisay et al., 2018). In some regions of SSA farmers also tried to control FAW using botanical pesticides such as ground chili pepper (*Capsicum annum* L.), tobacco extracts (*Nicotiana tabacum* L.), neem tree leaves (*Azadirachta indica* A. Juss.), and jatropha leaves (*Jatropha curcas* L.) (Baudron et al., 2019; Kumela et al., 2019). These botanical pesticides had traditionally been used to control other insect pests in field crops. They are cheaper alternatives for the resource‐poor farmers and are probably less hazardous to the farmers, environment, and non‐target insects (FAO, 2019b; Roman, 2016; Stevenson et al., 2017). Smallholder farmers in Mesoamerica have been reported to use the same practices in managing FAW (FAO,  2018a).

The relative effectiveness of some of these botanicals on FAW has been studied and reported (Babendreier et al., 2020; Hruska, 2019). Neem oil and neem seed and leaf powder were reported to have 70% mortality on FAW larvae (Maredia et al., 1992; Silva et al., 2015), and oil from Flooded gum (*Eucalyptus urograndis*) was effective in protecting maize (Hruska, 2019), while papaya (*Carica papaya* L.) seeds ground into powder were found to be as effective as the chemical insecticide malathion {diethyl 2‐[(dimethoxyphosphorothioyl)sulfanyl]butanedioate} (Archundia et al., 2006; Figueroa‐Brito et al., 2013). In Ghana, neem oil‐based products (0.17−0.33%) were found to be almost as effective as Emamectin benzoate (4′′‐Deoxy‐4′′‐epi‐methylamino‐avermectin B1) (Ema 19.2 EC) in reducing FAW damage in maize (Babendreier et al., 2020). In that study, both the low and high doses of the neem extracts had the same effect on FAW, hence lower doses were recommended for control.

Though most farmers in SSA are now aware of FAW (Hruska, 2019; Kansiime et al., 2019), the majority are not yet well equipped with knowledge and resources, to effectively control and sustainably manage the pest (Assefa & Ayalew, 2019; Hruska, 2019; Kumela et al., 2019). Kansiime et al. (2019) reported that farmers in Zambia used a total of 22 active ingredients in trying to control FAW, with some of the farmers mixing two to three insecticides in one spray to ensure effectiveness. Other farmers applied insecticides only once in the season, while some applied repeatedly but without a proper spraying schedule. Various rates were used on the basis of crop stage rather than insect stage (Kansiime et al., 2019). In Ethiopia, Assefa and Ayalew (2019) highlighted the fact that there were no registered insecticides for FAW, and that threshold levels were not being implemented to determine the need to spray.

A recent FAW population dynamics study in Ghana by Nboyine et al. (2020) recommended that farmers who used insecticides to control FAW needed to make the best use of the first 9 wk of crop establishment. These 9 wk mark the period in which the foliage of the maize crop will still be soft enough for young neonates, hence moths are attracted by the crop to lay eggs. High‐pressure insecticide spray directly on the whorl can give good chemical control, depending on the efficacy of the insecticide used and the larval stage of the FAW. However, the best results are achieved when control is instituted when the larva is young and before it burrows deep into the whorl or enters into the ears of the maize plant.

#### 3.2.2 Cultural agronomic practices

Yigezu and Wakgari (2020) summarized different cultural practices that have been utilized across SSA in managing and controlling FAW infestation and maize yield losses. These include handpicking and killing of larvae, placing sand or wood‐ash in whorls of maize plants, drenching plants with tobacco extracts, deep plowing to kill overwintering pupae, early planting, destruction of ratoon host plants, burning infested crop residues after harvesting, intercropping with non‐host plants, use of multiple cultivars, and rotation with non‐host crops (Kebede & Shimalis, 2019; Yigezu & Wakgari,  2020).

Studies by Baudron et al. (2019) reported the effectiveness of additional cultural practices, such as weeding and fallow periods against FAW for smallholder farmers in Zimbabwe. Results showed that repeated weeding reduced FAW damage, probably due to the fact that most of the weeds in the study area were FAW hosts of the Graminaceous family. Maize production under zero or minimum tillage was reported to reduce FAW damage in the Americas because it favored population build up for predatory species (Rivers et al., 2016). Kebede and Shimalis (2019) reported that conservation agriculture could reduce the impact of FAW through build‐up of natural enemies and boosting of the maize crops’ ability to fight the infestation. However, this contradicts the need to deep plow and burn crop residues in infested fields to control worms and pupae, as viewed by Yigezu and Wakgari (2020). Reduced and zero tillage are common practices in Africa; it is therefore important to do further research on this as an option of managing FAW in smallholder farmer systems in SSA.

However, maize intercropping with pumpkin (*Curcubita* spp.) increased FAW damage, most likely because pumpkin is a known host for FAW (Baudron et al., 2019). Intercropping of maize with legumes, such as cowpea [*Vigna unguiculata* (L.) Walp.], groundnut (*Arachis hypogaea* L.), and common bean (*Phaseolus vulgaris* L.), was also found to be ineffective in reducing FAW damage (Baudron et al., 2019). In contrast, Hailu et al. (2018), reported that intercropping maize with edible legumes significantly reduced FAW and stem‐borer infestation and damage. Yigezu and Wakgari (2020) also reported that non‐host legumes, such as bean, when intercropped with maize, significantly reduced FAW infestation and damage on maize. Altieri et al. (1978) reported the same findings in Colombia. Intercropping was also reported as one of the control strategies used by farmers in the Americas (FAO, 2017). It is believed that intercropping of two or more crops, or inclusion of non‐host crop plants in the field, can reduce FAW oviposition on the maize plant.

Traditionally, smallholder farmers in Central and South America apply sand or soil into the leaf whorls to control the pest. In southern Africa, some farmers used grains of sand, applied together with ammonium nitrate fertilizer, or as separate treatments to control FAW, and this significantly helped control the pest (Hruska, 2019). It is thought that sand scarifies the insect's body, predisposing it to infection by natural pathogens, while the ammonium nitrate fertilizer dehydrates the worm, thereby killing it. Kansiime et al. (2019) reported that about 19% of the surveyed farmers in Zambia used sand, ashes, and detergents to control FAW larvae during the 2016/2017 cropping season. The practice of spraying sugar solution and fish soup was also used by farmers in some parts of SSA (Harrison et al., 2019). The strategies are targeted at attracting and building up populations of natural enemies (predatory ants, parasitoids, solitary wasps, and other enemies) in the field.

General good crop management can help in managing FAW. The ability of the maize crop to withstand FAW attack is dependent on the nutritional and water status of the crop. Farmers in Zambia reported that FAW attack was more severe in fields where fertilizer was not applied compared with those where it was applied (Kansiime et al., 2019). The same farmers also reported that early planting and good timing of fertilizer application reduced FAW severity. A well‐fertilized and well‐watered crop can resist or recover rapidly and much better from FAW attack compared to a water‐stressed and nutritionally deficient maize crop. The maize crop can recover from low levels of FAW damage, however this is highly dependent on the crop's growth stage and nutritional status of the crop (Hruska,  2019).

According to Hruska (2019), cultural practices need to play a major role in FAW control in SSA. In view of the small land areas cultivated to maize by smallholder farmers in SSA, Hruska (2019) supported the idea that mechanical control strategies, such as egg squashing and larvae picking can work, citing experiences in Kenya and Ethiopia, where 337,000 and 402,000 ha were satisfactorily controlled in 2017 and 2018, respectively, using this strategy. Wightman (2018) considered the laying of eggs by FAW in large conspicuous clusters, a weakness of the pest itself, as this allowed for easy destruction of the pest through egg squashing or by predatory insects. Tambo et al. (2019) reported that larvae picking and chemical application were the major control strategies utilized in Ghana and Zambia, and a combination of the two practices gave the highest grain yield under FAW infestation. This was also supported by Kansiime et al. (2019), who reported that 36% of farmers in Zambia used cultural/mechanical methods to control FAW, and these were dominated by larvae picking and egg squashing. Physical squashing of larvae, moths, and egg masses and use of ashes and liquid detergents in FAW control were also noted in various other countries across Africa (Baudron et al., 2019; Rwomushana et al., 2018; Wightman, 2018).

#### 3.2.3 Biological control practices

The FAW has several natural enemies, such as predators, parasitoids, and pathogens that regulate its population levels. In some cases, intercropping creates an environment that favors development and growth of a population of natural enemies, large enough to control FAW (FAO, 2017). This observation has resulted in popularization of the “push–pull technology” (PPT) currently being recommended for FAW control (ICIPE, 2018). The method was developed by the International Centre of Insect Physiology and Ecology (ICIPE) for control of stem borers in maize and is now being promoted and used in several SSA countries to control FAW.

The PPT is based on intercropping maize with greenleaf desmodium [*Desmodium intortum* (Mill.) Urb.] and bordering the intercrop with *Brachiaria* 'Mulato II' (Midega et al., 2018). The *Desmodium* protects the maize by emitting semiochemicals that repel (push) the moths that are concurrently attracted (pulled) by semiochemicals released by the border crop. ICIPE (2018) and Midega et al. (2018) reported that FAW infestation can be reduced by at least 80% in a field where the technology is being practiced. Hailu et al. (2018), in their studies in Uganda, reported FAW infestation levels of 36−38% on maize under PPT, which were significantly lower compared to 95% infestation observed under sole cropping. Push–pull technology reduced FAW infestation in maize better than maize–legume intercropping (Hailu et al.,  2018).

Natural parasitism levels of more than 44% have been recorded in unsprayed fields in the Americas (FAO, 2017), which has implications for the development and recommendation of control strategies in SSA. A study by Sisay et al. (2018) in Ethiopia, Kenya, and Tanzania, identified five native species of parasitoids, some with parasitism levels as high as 45.3%. These include *Cotesia icipe* (Fernandez‐Triana & Fiobe), *Palexorista zonata* (Curran), *Coccygidium luteum* (Brulle), *Charops ater* (Szépligeti), and *Chelonus curvimaculatus* (Cameron). Several other studies have also identified natural enemies of FAW, with the same high levels of parasitism. Kenis et al. (2019) reported the presence of *Telenomus remus* (Dixon) in at least five SSA countries. Agboyi et al. (2020) observed 10 species of parasitoids (*T. remus*, *Chelonus bifoveolatus* (Szpligeti), *Trichogramma sp*., *C. luteum*, *C. icipe*, *Meteoridea* cf. *testacea* (Granger), *Charops* sp., *Metopius discolor* (Tosquinet), *Pristomerus pallidus* (Kriechbaumer), *Drino quadrizonula* (Thomson) in Benin and Ghana. In addition, Koffi et al. (2020) identified seven parasitoid species and three FAW predator species in Ghana. The parasitoids include *C. bifoveolatus*, *C. luteum*, *C. icipe*, *M. testacea*, *Bracon* sp., *Anatrichus erinaceus* (Loew), and an undetermined tachinid fly (Diptera: Tachinidae), while the predator species are *Pheidole megacephala*, *Haematochares obscuripennis*, and *Peprius nodulipes*.

Geographical variation in species occurrence and level of parasitism was noted (Sisay et al., 2018; Kenis et al., 2019; Agboyi et al., 2020; Koffi et al., 2020), which was due to differences in geographical areas, agronomic practices, crop type, and stage (Hay‐Roe et al., 2016; Ruíz‐Nájera et al., 2007). Further studies are required in this area to identify additional parasitoid species with high parasitism levels, to enable effective biological control in SSA.

In the Americas, mass rearing and release of parasitoids and predators are practiced, and are effective in managing FAW and other pests (FAO, 2018c; Parra & Zucchi, 2004; Soares et al., 2012). In SSA, classical bio‐control may need government intervention for implementation due to the high cost. However, in light of the recent studies performed in several SSA countries that have identified native parasitoids with good parasitism levels (Agboyi et al., 2020; Kenis et al., 2019; Koffi et al., 2020; Sisay et al., 2018), augmentative bio‐control becomes the most appropriate option. Unlike classical bio‐control, augmentative bio‐control involves periodic release of native species of the pest's natural enemies to augment natural biological control (FAO, 2018c). The Americas have found the parasitoid *Trichogramma* to be effective in controlling FAW through its attack on the pest's egg masses (Prasanna et al., 2018; Soares et al., 2012). Scientists at ICIPE in Kenya and Agboyi et al. (2020) also recommended the parasitoids, *Trichogramma* and *Telenomus*, for augmentative FAW bio‐control. Their research proved that the two wasps, when released into maize fields, search for and lay their eggs on the egg masses of FAW, thereby killing FAW before it has even hatched (ICIPE,  2018).

Entomopathogens are pathogens that affect insects and naturally regulate FAW populations in the Americas (Molina‐Ochoa et al., 2003). These have also been observed to control FAW in some farmers’ fields in Africa (FAO, 2018c). Assefa and Ayalew (2019) reported that FAW was susceptible, in the Americas, to 16 species of entomopathogens, mainly viruses and bacteria. They highlighted that the occurrence and distribution of bio‐control agents, including entomopathogens, was dependent on their habit and determined by geographical location, agricultural practices, and insecticide use. However, in SSA there is still need to investigate the presence and distribution of these entomopathogens for effective exploitation in FAW control strategies. The FAW larvae killed by viruses and fungi are easy to identify. Larvae killed by viruses become soft, usually with head hanging down from the leaves, while those killed by fungi become hard, appearing frozen on leaves with a whitish or light green color (FAO, 2018c). Farmers in Central America recycle fungal spores and viroid particles through spraying strained entomopathogen filtrate of larvae killed by viruses and fungi into the whorls of maize plants infested by FAW (FAO, 2018c). This promotes continued infection and death of FAW larvae by entomopathogens, which results in reduced FAW populations in maize fields. Virus‐based bio‐pesticides have also been used in the Americas (Valicente et al., 2010), but these may need testing and registration in SSA. Bio‐pesticides that have been tried and showed potential include viruses, such as nuclear polyhedrosis virus, bacteria, such as *Bacillus thuringiensis* (*Bt*) and fungi, such as *Metarhizium* and *Beauveria* spp. (FAO,  2017).

#### 3.2.4 Host plant resistance strategy

The United States uses both native and transgenic FAW resistance to manage FAW in maize, and transgenics have recorded the highest levels of resistance to the pest (Wightman, 2018; Williams &Davis, 1997). Native resistance is defined as resistance that is naturally available in the gene pool, harnessed through selection for effective use in agricultural production systems (Ni et al., 2014). Native resistance offers minimal but significant protection to a crop, but it is usually combined with other management measures in an IPM strategy. This strategy may work better for African farmers, who have limited access to finances to purchase chemical insecticides. Access to cultivars with some level of resistance or tolerance to FAW brings cost‐effective control to the resource‐poor smallholder farmers in SSA.

Native resistance has not been reported in SSA, because FAW is a new pest and no cultivars with native resistance have been released so far. A study in Zambia by Kansiime et al. (2019) reported greater use of improved cultivars compared to local cultivars, and this is true for most countries in southern Africa. Farmers in Zambia and Zimbabwe reported varying levels of variety susceptibility to FAW (Baudron et al., 2019; Kansiime et al., 2019), improved cultivars being more susceptible as opposed to open‐pollinated varieties (OPVs) and local cultivars (Kansiime et al.,  2019).

In Brazil, FAW was primarily controlled with insecticides until insecticide resistance became a problem, resulting in the introduction of *Bt* maize (Aguirre et al., 2016; Fatoretto et al., 2017; Sisay et al., 2018). The *Bt* maize has effectively managed FAW in the Americas (Womack et al., 2018) but with a 3−4 yr cycle of resistance breakdown (Fatoretto et al., 2017). Adoption of genetically modified (GM) maize in the United States, Brazil, and Argentina has surpassed 85% (Hruska,  2019).

Host‐plant resistance using transgenic cultivars is one strategy being used by farmers in South Africa (Botha et al., 2019). While most countries in SSA have policy restrictions on the use of GM crops (ISAAA, 2017), farmers in South Africa have been cultivating GM maize cultivars with insect‐resistance traits since 1998 (Kruger et al., 2012, 2014). The transgenic maize event MON810 was introduced for commercial production in South Africa in 1998 by the then Monsanto company (now Bayer), with the intention to control stem borer. However, the same genes also confer partial resistance to FAW (Prasanna et al., 2018). Another maize transgenic event, MON89034, was introduced in 2010, also by Monsanto (Bonsu & Esterhuizen, 2019). This event contains stacks of insect resistance traits; it is resistant to FAW, common Africa maize stalk borer, and spotted stalk borer. For now, the resistance is more durable compared to that in MON810, making it the most preferred maize event in controlling FAW in South Africa (ISAAA, 2017). Botha et al. (2019) reported moderately effective to highly effective field control of FAW with *Bt* events carrying stacks of Cry1A.105 + Cry2Ab2 proteins.

In general, transgenic cultivars carrying monogenic/oligogenic resistance normally exert high selection pressure on insect pests, potentially leading to the development of insect resistance (Hellmich & Hellmich, 2012; Kruger et al., 2012). Stem borer resistance to *Bt* maize was reported by Kruger et al. (2012) and this was thought to have been caused by poor compliance to refugia requirements. Puerto Rico, Brazil, and the United States have reported resistance of FAW to Cry1F *Bt* maize, while the same has been reported for Cry1Ab *Bt* maize in Brazil (Botha et al., 2019). Such cultivars, with complete insect pest resistance, should always be planted together with a refuge crop nearby to allow susceptible insect populations to survive and breed with the resistant pest (Fatoretto et al., 2017; Onstad, 2014a). A refuge crop is used to sustain a population of *Bt*‐susceptible pest strain (Botha et al., 2019; Kruger et al., 2014). Inter‐pest strain breeding between the *Bt*‐susceptible and *Bt*‐resistant strains can result in dilution of alleles for resistance, thereby slowing down insect pest resistance (Hurley & Mitchell, 2014; Mohankumar & Ramasubramanian, 2014; Onstad, 2014a). The refuge crops are used in South Africa by commercial farmers, who can afford using GM cultivars, and view the practice as more profitable than cultivation of non‐GM cultivars (Kruger et al., 2012). In the early years of the introduction of GM maize cultivars in South Africa, many commercial farmers, despite enjoying the benefits of growing GM maize cultivars, continued cultivating large areas of non‐GM maize for export to other African countries that preferred only GM‐free maize (Kruger et al., 2012). This implies that native resistance to FAW is the ultimate solution for most of SSA.

#### 3.2.5 Integrated pest management strategies

Following the invasion of SSA by FAW, key agriculture stakeholders in the region, including governments, international and non‐governmental organizations, discussed potential interventions. These included running widespread awareness campaigns, and training of farmers and agricultural stakeholders on building an IPM approach toward controlling and managing FAW (Day et al., 2017; Prasanna et al., 2018). An IPM strategy is based on the principle of controlling a pest using a combination of methods while causing the minimum possible damage to the environment, animals, and people. The IPM combines cultural, biological, host‐plant resistance, and safe pesticide control methods (Hurley & Mitchell, 2014; Onstad, 2014a, 2014b). The strategy conveniently aims at slowing down the indiscriminate use of pesticides, which many farmers were employing in trying to manage the pest.

The FAW IPM strategies are targeted at preventing or avoiding pest infestations, and management of established infestations. This involves routine scouting to identify and respond to infestations, to suppress the pest using the IPM triangle strategies, that is, minimum application of safe pesticides, provision of safe, scientifically proven or evidence‐based options to farmers, and managing insect resistance to pesticides (Onstad, 2014a; Prasanna et al., 2018). The IPM triangle is a practice that enhances effective application of IPM strategies by considering control as a three‐pronged strategy comprising of (a) chemical, (b) biological, and (c) cultural control, all based on effective pest monitoring (Onstad, 2014a; Zalom, 2010). The IPM triangle manages resistance through a concept of resistance management that ensures sustainability and eco‐friendly pest management strategies (Onstad, 2014a,  2014b).

Monitoring, scouting, and early detection are critical in controlling FAW. Pheromone traps are used to monitor the pest's presence and abundance in and around the field, and this can be used to forecast the pest's movements (Prasanna et al., 2018). The traps attract, trap, and kill male moths, but they do not sufficiently reduce the male moth population to disrupt mating, hence they should not be used for FAW control purposes (FAO, 2019b), but rather only for monitoring and forecasting. Effective monitoring and forecasting can only be achieved with highly specific cost‐effective lures that exclude non‐target species, otherwise low specificity leads to overestimation of infestation levels and false movement patterns (Meagher et al., 2019). Lures are synthetic compounds that mimic natural pheromones and these are put in traps to attract the moths (FAO, 2018d; Knodel et al., 1995). Studies conducted in the United States, Central America, and Brazil suggested that there were regional differences in pheromone attractiveness to FAW and also different pheromone‐lure compositions showed differential attraction for non‐target insects (Batista‐Pereira et al., 2006; Meagher, 2001; Meagher et al., 2013; Spears et al., 2016), and this is important for monitoring and surveillance in SSA. The FAO has reviewed the commercially available pheromone traps and has made recommendations on the best traps to use for FAW moths (FAO, 2019b). In Togo, Meagher et al. (2019) tested three commercial pheromone blends commonly used in the United States, together with two commercial and one locally made trap for optimal number of moths captured, lure specificity, and cost of monitoring. Regardless of lure type, commercial bucket‐type traps captured the most moths. The bucket‐3C lure trap combination showed the most desirable results, but it is relatively costly ($13.50 per trap), which makes the local‐3C trap combination a good and affordable ($3.50 per trap) choice for smallholder farmers in SSA, as it captured high numbers of moths (Meagher et al., 2019). Its low specificity could be compensated by placing more traps in the target area. Meagher et al. (2019) provide general guidelines regarding the most appropriate trap and lure type suitable for West Africa, but further studies are needed for other regions of SSA.

The FAW can have multiple generations in one field and different types of damage can be experienced simultaneously. Control using pesticides is most effective when the larva is still small and young (Nboyine et al., 2020; Prasanna et al., 2018), as older larvae may be difficult to control because of the “frass plug” that deters insecticide penetration into the larva (Bessin, 2004). The pest is usually more destructive on late‐planted crops, and in maize, it prefers the whorl stage (V2−V12 stages) (Bessin, 2004). Random sampling during scouting of the field to check for the pest or signs of infestation is key. It is recommended that at least 10− 20 consecutive plants should be checked daily for signs of infestation in a field, with the first plant chosen randomly (Bessin, 2004; Prasanna et al., 2018). For scouting to be informative, at least five different spots in a field should be sampled, conforming with FAO's “W” or “zig‐zag” sampling method (Bessin, 2004; FAO, 2018a). Different generations of larvae cause different types of injury to the plant (Harrison et al., 2019; Kumar, 2002). Correctly identifying the type of damage may help in determining the growth stage of the larvae, which can help in choosing an appropriate control strategy. Small larvae cause small “pin hole” and “window pane” damage on leaves, whereas the larger larvae prefer hiding in the whorl and cause foliage damage. During the flowering phase (VT stage = tasseling and silking), large larvae may be pushed out of the whorl by the tassel as it emerges and they may move to the ears for shelter and food (Bessin,  2004).

It is best for FAW control measures to be instituted when there is evidence of the pest's presence in the field (Onstad, 2014b; Prasanna et al., 2018; Stout, 2014). Effective control is realized when appropriate measures are implemented before damage reaches economic threshold levels (ETLs), that is, the pest population or pest damage that needs to be controlled so that the damage will not reach economic injury level (EIL) (Hunt et al., 1995; Paula‐Moraes et al., 2013). If the crop damage surpasses EIL, it will no longer be economical to control the pest. At the EIL, yield loss attributable to the pest is equal to the cost of controlling the pest (Paula‐Moraes et al., 2013). As a guide, chemical control strategies should be implemented when egg masses are spotted on at least 5% of the crop or when 25% of the crop at early whorl stage (or 40% at late whorl stage) is showing physical damage caused by the pest and when live pests are visible on the crop (Bessin, 2004; Prasanna et al.,  2018).

## 4 PROSPECTS FOR SUCCESS IN BREEDING FOR RESISTANCE TO FALL ARMYWORM

The most effective way to manage and control insect pests in crop production is to use insect‐resistant crops (Kumar, 2002; Rwomushana et al., 2018). Chemical control provides immediate control, but pesticide resistance is inevitable (Aguirre et al., 2016), and this led to the development of GM maize in the Americas (Aguirre et al., 2016; Gutirrez‐Moreno et al., 2019). Host‐plant resistance is a phenomenon that is expressed by the degree of the damage by the pest on the host plant. This is influenced by heritable characteristics encoded in the genome of the plant that enables it to suffer minimal damage by the pest (Mihm,  1997).

However, there has not been any systematic study in SSA that has determined and reported maize genetic resources resistant to FAW. Thus far, no research on breeding and release of FAW‐resistant maize genetic resources has been conducted in SSA (Prasanna et al., 2018). Reports from recent studies on other aspects of FAW have shown that maize cultivars grown in southern Africa and the SSA region, at large, have succumbed to the pest (Baudron et al., 2019; Kansiime et al., 2019). Various national programs, working together with CIMMYT and IITA, have initiated breeding programs for the development of FAW‐tolerant cultivars in SSA. In Zimbabwe, the national breeding program, collaborating with CIMMYT and local seed companies, has initiated screening of the diverse maize genetic resources and commercial cultivars. This strategy will help generate baseline information for advising farmers on cultivar use, or to be used by maize breeders to decide on best lines and/or populations for developing maize‐breeding populations for FAW resistance.

Breeding for insect pest resistance in maize started around the 1900s (Gernet, 1917; Hinds, 1914) and native FAW resistance was found in crops, such as maize, sorghum, millet, bermudagrass [*Cynodon dactylon* (L.) Pers.], and peanut (*Arachis hypogaea* L.) (Mihm, 1997; Stout, 2014; Wiseman & Davis, 1979). There have been considerable efforts to breed maize for native resistance or tolerance to FAW in the Americas (Mihm, 1997; Mihm et al., 1988; Williams et al., 1983; Wiseman et al., 1981), and successes have been reported in literature (Brooks et al., 2005; Kumar, 2002; Stout, 2014). Unfortunately, native breeding for FAW has been quickly overtaken by the advent of *Bt* maize (Hellmich & Hellmich, 2012; Wightman, 2018; Xiao & Wu, 2019). In the 1970s–1990s, CIMMYT, the USDA‐ARS, the Brazilian Agricultural Research Corporation, EMBRAPA, and several U.S. universities identified and developed several improved temperate, tropical, and subtropical maize materials with at least partial resistance to FAW (Kumar, 2002; Mihm, 1997; Ni et al., 2008). These materials have been used to breed for resistance to FAW in the Americas, and are potential sources of genes for resistance to FAW, which can be introgressed into current SSA‐adapted maize lines, for developing locally adapted hybrids resistant to FAW.

Some of these genetic resources have been introduced into several SSA countries, including Ethiopia, Kenya, Nigeria, and Zimbabwe, and crossed to locally adapted lines (Prasanna et al., 2018). New cultivars can be developed through introgression of the FAW‐tolerance genes into locally adapted but FAW‐susceptible inbred lines, as Womack et al. (2018) suggested. Zimbabwe's national maize‐breeding program, collaborating with CIMMYT and the International Atomic Energy Agency, carried out mutation induction in locally adapted materials. New inductions were targeted on selected FAW‐tolerant donor lines. It is anticipated that novel lines may be selected for effective breeding of FAW tolerance in the SSA region, potentially leading to production of hybrids for testing and release. Newly developed maize cultivars are often disliked by farmers on account of taste issues (Wightman, 2018). It is therefore very important to include farmer preferences when developing and testing new FAW‐tolerant cultivars.

Across the years, plants have developed ways to resist/tolerate insect pest herbivory, and these include morphological, biochemical, and molecular mechanisms (Womack et al., 2018). Several scientists have classified host‐plant resistance into three different categories, which are non‐preference, antibiosis, and tolerance (Stout, 2014). Previous research has shown that antibiosis is the major mechanism responsible for FAW resistance in resistant genotypes (Williams & Davis, 1997; Wiseman et al., 1981). Non‐preference is the mechanism that confers resistance, mainly on account of hairs on the leaves and stems, thick leaf cuticles, and the shiny leaf texture (Mihm, 1997). Ni et al. (2008) investigated the resistance of maize inbred lines to both foliar and ear damage by FAW. Included in the study were four CIMMYT inbred lines (CML333, CML335, CML 336, and CML338) with varying levels of silk maysin that confers resistance to corn earworm (*Helicoverpa zea*). Earworm is a close relative of FAW. The study concluded that earworm‐resistant maize inbred lines with varying levels of silk maysin could confer cross‐resistance to foliage‐feeding FAW at the seedling stage (Ni et al., 2008). The CIMMYT maize line (CML), CML338, was among the inbred lines with resistance to earworm (Ni et al.,  2008).

**FIGURE 3:**
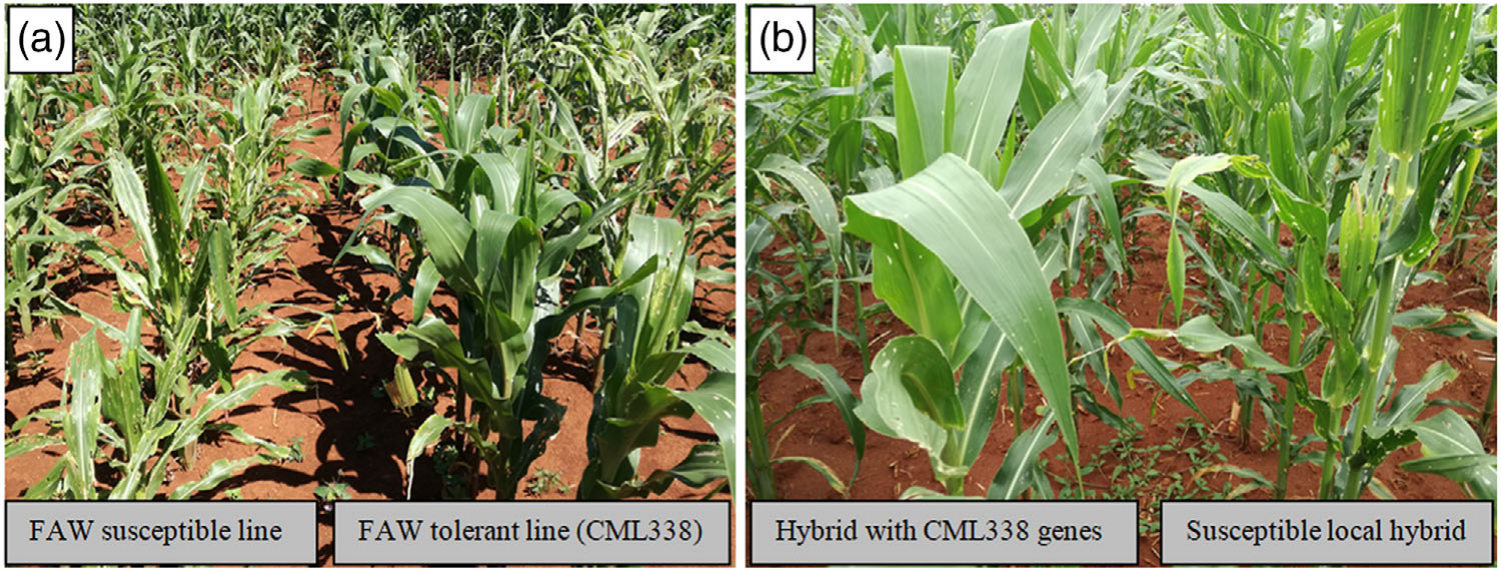
(a) Fall armyworm (FAW) tolerance donor line, CML338, that resists foliar damage by FAW, compared to a susceptible local line, in a field planted under natural infestation in Harare, and (b) A susceptible local hybrid compared side by side with a hybrid with introgressed genes for FAW tolerance from CML338 under natural FAW infestation in Harare in March 2020

The CIMMYT lines, CML338 (Figure 3a), CML67, and CML139 (of Antiguan origin) (Kumar, 2002; Mihm et al., 1988), are among the CIMMYT FAW‐tolerance donor lines that were introduced into SSA for the purpose of introgression of FAW‐resistance genes into the locally adapted elite inbred lines to develop FAW‐resistant, locally acceptable inbred lines for hybrid development. The hybrids CML67 and CML139 possessed non‐preference and antibiosis resistance that was heritable in crosses between susceptible and resistant lines (Kumar, 2002; Kumar & Mihm, 1996). Breeding crosses that have been made so far with FAW‐tolerant donor lines and local lines in eastern and southern Africa are yielding promising resistant genotypes (see Figure 3b), and this gives hope for success in breeding locally adapted cultivars that have acceptable resistance to FAW.

In resistance breeding, in general, partial resistance is usually preferred to complete resistance, as partial resistance confers horizontal resistance, which is more durable and takes longer to break down as the pest continues to evolve across time (Chandrasena et al., 2018; Farias et al., 2014; Fatoretto et al., 2017; Storer et al., 2010). In most cases, native resistance in maize has been found to be polygenic and quantitative in nature and is durable compared to monogenic and oligogenic resistance, typical of transgenics (Chandrasena et al., 2018; Huang et al.,  2014).

Fall armyworm resistance is determined on the Davis scale, which measures the extent of damage to foliage or ear on a scale of 1 (most resistant) to 9 (most susceptible) (Davis et al., 1992; Davis and Williams, 1994; Huang et al., 2014). Maize cultivars exhibiting multiple‐gene resistance (partial resistance) to FAW, have resistance scores from 3 to 5 on the Davis scale. These differ from their genetically modified counterparts carrying Lepidopteran monogenic or oligogenic resistance, which exhibit scores of 1–2 on the Davis scale (Aguirre et al., 2016). It is therefore important for breeding programs to consider durability of resistance as much as the source and strength of the resistance. This is particularly important in Africa, where variety replacement is still slow. African farmers would need a variety that has durable FAW resistance, which they can use for several years without resistance breakdown.

Multiple trait resistance attributable to stacking of native resistance genes or trans‐genes into locally adapted maize cultivars with native resistance, can increase durability of FAW resistance (Huang et al., 2011; Prasanna et al., 2018). This works better if breeders combine several traits with different modes of action or toxic proteins (Huang et al., 2014; Fatoretto et al., 2017). Previous research in the Americas has confirmed that stacking multiple insect‐resistance genes effectively controls FAW and manages insect resistance much better (Fatoretto et al., 2017; Mohankumar & Ramasubramanian, 2014). In South Africa, Botha et al. (2019) reported high levels of FAW mortality (>99%) with the pyramid toxic event Cry1A.105 + Cry2Ab2 in tolerant and resistant genotypes. Baudron et al. (2019) and Kansiime et al. (2019) reported varying levels of response of different genotypes to FAW infestation in Zambia and Zimbabwe. This creates prospects of improving further maize cultivars for tolerance to FAW.

South Africa has been partnering with Kenya, Mozambique, Tanzania, and Uganda in the water‐use efficient maize for Africa (WEMA) project targeted at developing and disseminating improved maize cultivars for the smallholder farmers (Prasanna et al., 2018). The project was initiated in 2012 and it tested the *Bt* maize event MON810 and other locally adapted maize genotypes that had been stacked with *Bt* and drought‐tolerance genes (DT; DroughtGard (Bayer) or CspB from *Bacillus subtilis*). The cultivars have been tested for safety and efficacy of the transgenic traits against Lepidopteran pests, such as stem borer and FAW, and drought stress under confined environments (ISAAA, 2017). Results from the WEMA project in Kenya, Uganda, and Mozambique have shown that introgression of MON810 into locally adapted maize cultivars confers strong stem borer resistance and partial FAW control in maize (Prasanna et al., 2018). Use of these *Bt* events in countries that allow cultivation of GM maize can be a good FAW‐management option for the farmers. However, there are significant differences in the status of the biosafety systems in different African countries, with South Africa being one of the few countries having an established biosafety system in place. The African Biosafety Network of Expertise (ABNE) was established to enhance the capacity of African countries to build functional biosafety regulatory systems (ABNE,  2020).

However, studies by Botha et al. (2019) suggest that the FAW introduced in South Africa and probably most of SSA might have resistance alleles against Cry1Ab or the event is a low dose for FAW control. This implies that Cry1Ab *Bt* maize events, such as MON810, require gene stacking for effective FAW control. Gene stacking will also help slow down resistance development regarding the FAW pest. In SSA, the majority of smallholder farmers cannot afford to purchase *Bt* cultivars, as they are usually costly. In addition, there is always a refuge crop nearby under smallholder farmer systems. However, development of FAW resistance to present and future *Bt* maize cultivars remains a threat; therefore, insect resistance‐management strategies should be put in place. Botha et al. (2019) suggested to begin with a baseline susceptibility study for FAW, as it will guide future assessments. In contrast, Wightman (2018) doubts the feasibility of use of *Bt* maize in SSA, citing seed cost issues against the low producer price of maize characteristic of the SSA market.

Host‐plant resistance is an integral part of an IPM strategy (Stout, 2014; Zalom, 2010). It is environmentally friendly and cost effective, as it reduces production costs by reducing the cost of insecticides (Mohankumar & Ramasubramanian, 2014; Womack et al., 2018). It is of paramount importance that breeders, in their efforts in developing FAW‐resistant cultivars, consider managing insect pest resistance. CIMMYT and its partners reviewed germplasm known to have resistance to FAW, and tagged those as potential sources of FAW‐resistance genes, and concluded that there was enough diversity and that conventional breeding could support effective FAW‐resistance breeding in SSA (Prasanna et al., 2018). Several breeding programs in SSA have embarked on intensive FAW‐resistance breeding (Kasoma et al., 2020; Prasanna et al., 2018). Mutation induction and conventional crosses are being employed to create and introgress new alleles for FAW resistance into locally adapted maize materials. The focus is on developing elite hybrids that combine FAW resistance with farmer‐ and industry‐preferred traits.

At present, in most of these efforts, selection is conducted mainly under natural infestations, as there are few insect‐rearing and screen‐house facilities for artificial infestation trials in most countries, an exception being CIMMYT in Kenya and perhaps a few private seed companies. Testing protocols for screening cultivars and inbred lines under natural FAW infestations have been developed and well documented, and these can provide effective screening (Prasanna et al., 2018). In addition, efforts are underway at CIMMYT in Zimbabwe and probably other countries, to develop insect‐rearing and testing facilities for selection of lines and cultivars with resistance to FAW. Womack et al. (2018) recommended the use of molecular markers to aid phenotypic selection for FAW resistance. The latter is believed to effectively achieve genetic gains in the shortest time. This implies that selection pressure will be applied only to validate selections done through marker‐assisted selection. However, this option will only be feasible upon release of validated markers for FAW‐resistance selection in maize. Fall armyworm resistance is polygenic, but no quantitative trait loci studies for FAW resistance has been done in African maize germplasm yet (Badji et al., 2018). Currently, genome‐wide association studies (GWAS) is the most advanced strategy for mapping of genome regions associated with traits of interest, such as FAW resistance (Chakradar et al., 2017). The first GWAS study for FWA and maize weevil (MW) resistance in African maize germplasm was carried out by Badji et al. (2020), in a diverse association mapping panel of maize inbred and doubled haploid lines developed in a wide range of African agro‐ecologies. They found 62 quantitative trait nucleotides (QTNs) on all 10 maize chromosomes which were associated with FAW and MW resistance of which six were associated with both FAW and MW resistance, showing pleiotropic genetic control of resistance to these pests. Marker‐assisted selection strategies for FAW resistance are therefore still in development.

The WEMA project demonstrated that stacking of *Lepidopteran* spp. resistance traits into locally adapted materials can improve FAW resistance (ISAAA, 2017; Prasanna et al., 2018). Effectively, it means that introgression of native FAW‐resistance genes into SSA locally adapted materials can create sustainable and policy‐acceptable FAW control. This creates the potential to develop lines and cultivars (hybrids and OPVs) that combine high grain yield potential and FAW resistance. CIMMYT is in the process of converting preferred but FAW‐susceptible lines into resistant lines, and 10 promising CIMMYT maize inbreds have been identified and validated in Kenya (Rwomushana et al.,  2018).

## 5 CONCLUSIONS

Fall armyworm is difficult to control, manage, or eradicate, because it is polyphagous and trans‐boundary, has high multiplication capacity and a short life cycle, harbors a high migratory capacity through trade and natural winds, and lacks the diapause phase in its growth. An IPM control strategy, guided by cultural approaches already being used by farmers and what can be adopted from the Americas, coupled with an insect resistance‐management strategy, is the best option to manage this pest in Africa. These strategies will be strengthened by breeding for multi‐trait host‐plant resistance through stacking of genes for different modes of control of the pest. Maize‐breeding teams in SSA need to evaluate new and old commercial cultivars, breeding populations, and lines for FAW resistance. CIMMYT and several international research organizations have developed FAW‐resistant lines. These FAW‐resistance trait donors should be introduced into SSA maize‐breeding programs for trait introgression into the locally adapted lines with or without native FAW resistance genetic backgrounds. Mutation breeding is another option that can be used to enhance FAW tolerance and improve agronomic performance in donor lines. Going forward, it may be important for all breeding programs to consider releasing maize cultivars with a base line tolerance to FAW. In developing such cultivars, breeders need to introgress both FAW‐resistance genes and good agronomic traits for high grain yield to bring effective genetic gains to the farmers.

## CONFLICT OF INTEREST

The authors declare no conflict of interest.
